# TRIM21 Restricts Coxsackievirus B3 Replication, Cardiac and Pancreatic Injury via Interacting With MAVS and Positively Regulating IRF3-Mediated Type-I Interferon Production

**DOI:** 10.3389/fimmu.2018.02479

**Published:** 2018-10-25

**Authors:** Hui Liu, Min Li, Yahui Song, Wei Xu

**Affiliations:** Jiangsu Provincial Key Laboratory of Infection and Immunity, Institute of Biology and Medical Sciences, Soochow University, Suzhou, China

**Keywords:** TRIM21, coxsackievirus B3 (CVB3), viral myocarditis, IFN-β, IRF3

## Abstract

Tripartite motif-containing 21 (TRIM21) is a regulator of tissue inflammation and pro-inflammatory cytokine production, and has been implicated in negative regulation of IRF3-dependent type I interferon signaling. However, the antiviral activity of TRIM21 varies among diverse viruses and its role on regulation of type I interferon remains inconsistent in different microbial infections. Here, we investigate the potential role for TRIM21 in controlling Coxsackievirus B3 (CVB3) replication and susceptible organ pathology. We found that CVB3 infection up-regulated the expression of TRIM21 in hearts of mice and cardiomyocytes at early phase of infection. Knock-down of TRIM21 resulted in increased viral replication, while overexpression led to increased phosphorylation and dimerization of IRF3, increased IFN-β transcription and reduced viral replication *in vitro*. We demonstrate that TRIM21 promotes the activation of IRF3 in CVB3-infected cells via interacting with MAVS and catalyzing the K27-linked polyubiquitination of MAVS, thereby enhancing type I interferon signaling. The RING domain of ubiquitin ligase activity and PRY-SPRY domain of TRIM21 are critical for its anti-viral effect. *In vivo* overexpression of TRIM21 significantly protected mice against viral myocarditis by suppressing CVB3 replication and reducing cardiac inflammatory cytokine production. While TRIM21 deficient mice exhibited a decreased IFN-β production, an increased cardiac and pancreatic CVB3 replication, and aggravated pancreatic injury as well as myocarditis during acute infection. Thus, our results demonstrate TRIM21 as a positive regulator of IFN-β signaling by targeting MAVS during CVB3 infection and suggest it as a potent host defense against CVB3 infection and viral-induced injury in hearts and pancreas.

## Introduction

Coxsackievirus is a single-Stranded RNA non-enveloped virus of the Enterovirus genus within Picornaviridae associated with several human and mammalian diseases, of which B3 type Coxsackievirus (CVB3) is well-identified as a major causative agent of viral myocarditis (VMC) (1, 2). CVB3 has been involved in 25–27% cases of acute myocarditis and dilated cardiomyopathy in children and young adults (3). VMC has been identified as an important etiology of heart failure and dilated cardiomyopathy, which contribute to nearly 50% of the indication for the heart transplantation (4). CVB3 infection also involves brain and pancreas, resulting in aseptic meningitis and pancreatitis (5, 6). Early direct virus-induced cytopathic effect and intense inflammatory injury followed by host immune responses are the main pathological processes of VMC and pancreatitis. Although excessive activation of immune response triggered by virus infection maybe a major factor contributing to tissue injuries, the virus itself is critical to the progression of VMC via direct attack on cardiomyocytes (7, 8). Despite considerable effort for decades, the fundamental mechanism responsible for the pathogenesis of viral myocarditis has not been well-understood and no effective therapies for VMC are currently available. During acute phase, CVB3 replication leads to myocardial and pancreatic injury directly through inducing apoptosis and necrosis of cardiomyocytes and pancreatic acinar cells. CVB3 RNA can be detected in the chronic stages in infected animals by 21 days post-infection, initiating the disease progression to more severe myocardial fibrosis and DCM (8, 9). In this sense, development of novel anti-viral compounds and early intervention represents an alternative way to treat CVB3 myocarditis and related cardiomyopathy.

The tripartite motif (TRIM) protein family contains over 70 members of TRIM protein family in human and is structurally characterized by a RING domain, one or two B-boxes, and a coiled-coil domain (10). TRIM proteins have been reported to be involved in multiple biological processes including the regulating innate immunity, carcinogenesis, cell differentiation and apoptosis, which are mainly dependent on the RING domain of ubiquitin ligase activity and B-box domain of interacting motif (11, 12). Recently, a growing body of evidence suggests that many TRIM proteins play important roles in direct antiviral activities and in the regulation of antiviral innate immunity. TRIM5α was found to inhibit HIV-1 replication by directly interacting with viral proteins (13). TRIM22 has been reported to exert antiviral activity against several viruses, such as hepatitis B virus (HBV), encephalomyocarditis virus (ECMV), and human immunodeficiency virus (HIV-1) (14–16).

TRIM21, initially known as an autoantigen Ro52/SS-A, is an ubiquitously expressed cytosolic E3 ubiquitin ligase and plays important roles in immune regulation and microbial restriction. TRIM21 has been well known as a regulator for type I interferon (IFN) production, however it may positively or negatively modulate the antiviral innate signaling according to the types of viruses. TRIM21 has been reported to be a positive regulator of IRF3 signaling by preventing its ubiquitination and degradation, thus enhancing IRF3 mediated antiviral responses (17).On the other hand, Higgs et al. claimed that TRIM21 catalyzed IRF3 ubiquitination and promoted its degradation leading to inhibition of interferon-β (IFN-β) production post-pathogen recognition (18). TRIM21 also serves as a negative regulator of IFN-β during Japanese encephalitis virus (JEV) infection in human microglial cells (19).Recently, Xue et al. report that TRIM21 is upregulated upon RNA virus infection and promotes K27-linked polyubiquitination of MAVS to upregulate type-I interferon signaling, thereby inhibiting viral infection (20). Thus, the antiviral activity of TRIM21 varies among different viral infection. Up to the present, there is no report of TRMI21 on CVB3 infection; and almost no biological function of TRIM21 has been confirmed in animal models of viral infection. It is of great interest to explore the possible antiviral function of TRIM21 on CVB3 infection and its role in the disease progression of CVB3-induced VMC and pancreatitis.

Here, we investigate the antiviral activity of TRIM21 against CVB3 replication and its role in CVB3-induced acute vial myocarditis and pancreatic injury. Our results indicate that TRIM21 inhibits CVB3 replication via interacting with MAVS for promoting the K27 polyubiquitination of MAVS, thereby enhancing IRF3-mediated type I IFN signaling pathway and protecting mice against CVB3-induced myocarditis as well as pancreatic acinar cell necrosis.

## Materials and methods

### Mice and virus

Six–eight weeks old male BALB/c mice were purchased from the Shanghai Slac Animal Inc. TRIM21^−/−^ mice were constructed from Cyagen Biotech (Guangzhou, China).CVB3 (Nancy strain) was a kind gift from Professor Yingzhen Yang (Key Laboratory of Viral Heart Diseases, Zhongshan Hospital, Shanghai Medical College of Fudan University).

### Cell culture

HeLa cells and HEK-293 cells were grown and maintained in DMEM medium supplemented with 10% FBS (Gibco) and 100 units/ml penicillin and streptomycin in a 5% CO_2_ incubator at 37°C.

### Virus titers assays

The viral titer was determined by TCID_50_ assay on HeLa cell monolayers with standard methodology (AdEasy Application Manual, version 1.4; Qbiogene, Carlsbad, CA, United States).Cell culture and tissue lysis supernatants were diluted serially using 10-fold dilutions and titered on HeLa cell monolayers by the TCID_50_ assay.

### Plasmids and transfection

The Flag-MAVs or HA-K27Ub plasmids were the gifts from Prof. Hui Zheng (Institute of biological and medical sciences, Soochow University). Human TRIM21 cDNA was amplified from RNA of HeLa cells using primers: For: 5′-GCCACCATGGATTACAAGGATGACGACCGATAAGGCTTCAGCACGC-3′ and Rev: 5′-AAAGCCATCAATAGTCAG-3′. Mouse TRIM21 cDNA was amplified from RNA of cardiomyocytes using primers:5′-ATGGATTACAAGGATGACGATAAGCACCCTCTACAACCTCAAAA-3′ and 5′-CCTGGCTCCTGACCATCACA-3′. cDNA of truncated forms of TRIM21 lacking N-terminal RING,B-box C-terminal and PRY-SPRY domain were amplified using primers: ΔRING-For: 5′-CCGGCAGCGCTTTATGCTGCTC-3′ and ΔRING-Rev: 5′-AAAGCCATCAATAGTCAG-3′; ΔB-box-For: 5′-ATGCGGTGTGCAGTGCATGGA-3′ and ΔB-box-Rev: 5′-AAAGCCATCAATAGTCAG-3′; ΔPRY/SPRY-For: 5′-GCCACCATGGCTTCAGCAGCACGC-3′ and ΔPRY/SPRY-Rev: 5′-TCACACATGGCACACACTC-3′. For the transfection experiment, HeLa cells were seeded into 24-well plates and transient transfection was performed by Lipofectamine 2000 according to the manufacturer's instruction. Cells were cultured for 24 h before infection of CVB3.

### Quantitative real-time PCR (Q-PCR)

Total RNA was isolated from cells or tissues using RNAiso reagent (Takara, Cat. No. 9109), and cDNA was prepared using reverse transcriptase (Takara, Cat. No. DRR063A). Quantitative real-time RT-PCR (Q-PCR) was performed using SYBR green real-time PCR kits (TaKaRa, Cat. No. DRR041A)on a Bio-Rad iCycler using the following primers:

**Table:** 

	For (5′-3′)	Rev (5′-3′)
human-TRIM21	TGGTGTGTGCCC AGTCT	CATCGTGAGATCCAT TTCCA
mouse-TRIM21	AGGGTTAGAGGGGC TGTGTT	GACCATGGCTCCCTC ATCTA
human-IFN-α	GCCTCGCCCTTTG CTTTACT	CTGTGGGTCTCAGGG AGATCA
human-IFN-β	ATGACCAACAAGTGT CTCCTCC	GCTCATGGAAAGAGC TGTAGTG
mouse- IFN-β	CCCTATGGAGATG ACGGAGA	CTGTCTGCTGGTGGA GTTCA
ISG15	TCCTGGTGAGGAATAAC AAGG	GTCGTCGTCAGCCA GAACAG
ISG54	ATGTGCAACCTACTG GCCTAT	TGAGAGTCGGCCCAT GTGATA
human-GAPDH	CATGAGAAGTATGACA ACAGCCT	AGTCCTTCCACGATAC CAAAGT
mouse-GAPDH	TGGATTTGGACGCAT TGGTC	TTTGCACTGGTACGT GTTGAT

The 2^−ΔΔCT^ method was used to normalize the transcription of the detected gene mRNA to that of the GAPDH mRNA and calculate the fold induction relative to the control.

### Short interference RNA (siRNA)

Human siRNA oligonucleotides targeting sequences named as TRIM21 siRNA1 (UCAUUGUCAAGCGUGCUGC) and TRIM21 siRNA2 (UGGCAUGGAGGCACCUGAAGGUGG) were ordered from GenePharma.Inc (Shanghai, China). The siRNA was transfected into HeLa cells using INTERFERin *in vitro* siRNA transfection reagent (Polyplus, NewYork, United States).

### Western blotting

HEK293 cells were transfected with plasmids containing human or murine TRIM21 (1 μg) using the Lipofectamine Plus reagent (Invitrogen) for 24 h, and then infected by CVB3 (MOI = 5) for 18 h. Samples were resuspended in sample lysis buffer (Bio-Rad). Lysates were resolved by SDS-polyacrylamide gel electrophoresis and transferred to PVDF membranes. The blots were probed primary antibodies for Flag (1:1,000, CST 8146S), IRF3 (1:1,000, CST, D8389), pIRF3 (1:1,000, CST, S396), actin (1:2,000, ABGEN, SG140609AB), GAPDH (1:10,000, Sigma, G9545), and VP-1 (1:2,000, Dako, M706401). HRP-conjugated anti-rabbit (1:4,000, CST, 7074) or anti-mouse IgG (Bioworld, AB54151) was used as a secondary antibody. Proteins were detected by chemiluminescence (Pierce). The intensities of the bands in the blots were quantified by densitometry using the Image Studio Lite program according to the developer's instructions.

### IP and immunoblotting

HEK293 cells were transfected with an expression plasmid encoding full-length of Flag-tagged MAVS. Cell lysates were collected using radioimmunoprecipitation assay (RIPA) lysis buffer with protease inhibitors (1 mM phenylmethylsulfonyl fluoride, Roche complete protease inhibitor), followed by immunoprecipitation with anti-Flag beads. Proteins were eluted from the beads after washing six times with PBS. The protein binding to the beads was subjected to Western blot with anti-TRIM21 (1:2,000 Santa Cruz Biotechnology, SC25351) or anti-Flag (1:1,000, CST 8146S).

### Ubiquitination assays and native page

For analysis of ubiquitination of MAVS in HeLa cells, cells were co-transfected with TRIM21, HA-K27ub or Flag-MAVS, followed by infection with CVB3. Cell lysates were immunoprecipitated with anti-Flag and analyzed by immunoblotting with the anti-HA antibody. Native page for the detection of IRF3 dimerization was performed on acrylamide gel without SDS. Cells were lysed with ice-cold lysis buffer including 50 mM of Tris-Hcl at PH = 7.5, 150 mM of NaCl and 0.5% NP-40 containing protease inhibitor cocktail. After centrifugation at 13,000 g for 15 min, proteins in the supernatant were quantified and diluted with 5x native PAGE sample buffer (312.5 mM Tris-HCl, pH = 6.8; 75% glycerol; 0.25% bromophenol blue). The gel was pre-run for 30 min at 40 mA on ice with 25 mM Tris-HCL (pH = 8.4), and 192 mM glycine with or without 1% of deoxycholate in the cathode chamber and anode chamber, respectively. The unboiled total protein was added into the gel for 80 min at 25 mA on ice.

### Luciferase reporter assay

HEK293 cells were co-transfected with 100 ng luciferase reporter plasmid, 10 ng thymidine kinase promoter-Renilla luciferase reporter plasmid, and the TRIM21-expression or control vector plasmid using the Lipofectamine 2000 transfection reagent (Invitrogen,Cat.No.116688-019). 48 hrs later, cell lysates were prepared and the luciferase activities were determined by the Dual-Luciferase Reporter Assay System (Promega,Cat.No.E10910) according to the manufacturer's instructions.

### CVB3 infection

Mice were infected intraperitoneally (i.p.) with 100 μl PBS containing 1000 TCID_50_ dose of CVB3. Body weight and mortality of mice were recorded upon the termination of experiment. Individual experiments were conducted at least three times with 7 to 10 mice per group.

### Histopathological analysis

Three hearts and pancreas of each group of mice were collected 7 days post infection. The apical parts of the tissues were fixed in 10% phosphate-buffered formalin, embedded in paraffin wax, sectioned at 5 μm and stained with hematoxylin–eosin (H&E). Stained sections were used for image analysis with a Nikon Eclipse TE2000-S microscope and five images were captured under high power fields randomly.

### Immunohistochemistry

Hearts were fixed with 10% formalin in 0.1 M phosphate buffer, pH 7.4. Sections were deparaffinized and irradiated at 750 W in a microwave oven in 10 mM sodium citrate buffer, pH 6.0. Sections were then treated with 3% hydrogen peroxide to inhibit endogenous peroxidases. After washing in TBS with 0.025% Triton X-100, the sections were blocked with 10% BSA. Following blocking, sections were incubated with goat polyclonal antibody against TRIM21 (sc-21362; 1:500; Santa Cruz Biotechnology) diluted in TBS-1% BSA overnight at 4°C. After washing, sections were incubated with a biotinylated anti-goat secondary antibody (Jackson immunoresearch) for 1 h and a peroxidase-labeled streptavidin for 5 min at room temperature. Peroxidase activity was detected with DAB (Mouse and Rabbit Specific HRP/DAB Detection IHC Kit, ab64264, Abcam), and sections were counter stained with hematoxylin. The level of protein accumulation was estimated as the percentage of the total counterstained area that was positively stained for the protein of interest, which was determined using Image Jsoftware (Nikon Eclipse TE2000-S microscope).

### Primary cardiomyocyte culture

Neonatal cardiomyocytes were isolated from 1 to 3 days BALB/c mice. The ventricles obtained from 1 to 3 days BALB/c mice were removed rapidly into cold Hanks' balanced salt solution (Gibco). After washing and mincing, tissues were digested in 0.05% trypsin (Gibco) for 30 min at 4°C with rotation before transfer into DMEM (Gibco) containing 20% FBS (Fetalbovine serum, Gibco) to terminate the digestion. After washing with HBSS, the tissues were incubated with Liberase TH (0.1 U/mL, Roche, Germany) at 37°C for 5 min, and the dissociated cells were collected into 20% FBS DMEM. This procedure was repeated until most of the cells were released. The isolated cells were incubated with 5% CO_2_ at 37°C for 2 h. The unattached cardiomyocytes were seeded into fibronectin-coated 8-well Live Cell Imaging Culture Dish (Bestmagsystem Medical Co. Ltd., Suzhou, China) and experiments were performed when the cardiomyocytes formed a confluent monolayer and beat in synchrony at 72 h.

### Immunofluorescence

Immunofluorescence was performed to assess the cellular expression and location of TRIM21 and viral RNA expression according to the manufacturer's instructions. Freshly cultured cardiomyocytes were infected with CVB3 (MOI = 5) for 0, 12, and 24 hrs. Heart tissues of infected mice were embedded in OCT and made into 5 μm cryo section. Cells were fixed with 4% paraformaldehyde for 1 h before blocking (2% BSA) for 1 h. Cells were then incubated with primary antibodies against TRIM21 (1:200, Santa Cruz Biotechnology) and anti-dsRNA (1:300, J2 mAb, English and Scientific Consulting) at 4°C overnight. Fluorochrome-conjugated secondary antibodies (1:200, goat anti-rabbit IgG, goat anti-mouse IgG, Southern Biotech) and DAPI were used for immunofluorescent staining. Images were captured and analyzed with Nikon A1 confocal microscope.

### Enzyme-linked immunosorbent assay (ELISA)

Levels of TNF-α, IL-6, IL-10, IFN-γ, MCP-1 were determined using sensitive mouse, IL-6, IL-10, and IFN-γ kits according to the manufacturers' instructions (eBioscience, San Diego, USA).

### *In vivo* overexpression of TRIM21

Mice were retro-orbitally injected with 1.0 ml reagent containing 50 μg of mouse TRIM21-expression plasmid or vector plasmid using *in vivo*-JetPEITM–Gal transfection agent according to the manufacturer's instructions (Polyplus-transfection Inc., USA). Mice received 2 doses of TRIM21 plasmids 2 days before and 1 day after CVB3 infection to sustain *in vivo* over-expression of TRIM21.

### Statistical analysis

Data were presented as the mean ± SEM and statistical analysis was analyzed by GraphPadPrism 5 software. For two-group comparisons, statistical significance was determined by Student's *t*-test. Survival curves were estimated from Kaplan-Meier procedure with the Lonrank test to compare survival among groups. *P* < 0.05 was considered to be statistically significant and are indicated as follows: ^*^, 0.05 ≥ *P* > 0.01; ^**^, 0.01 ≥ *P* > 0.001; ^***^, *P* ≤ 0.01.

## Results

### TRIM21 is up-regulated in hearts of mice and in the murine cardiomyocytes upon CVB3 infection

**Figure 1:**
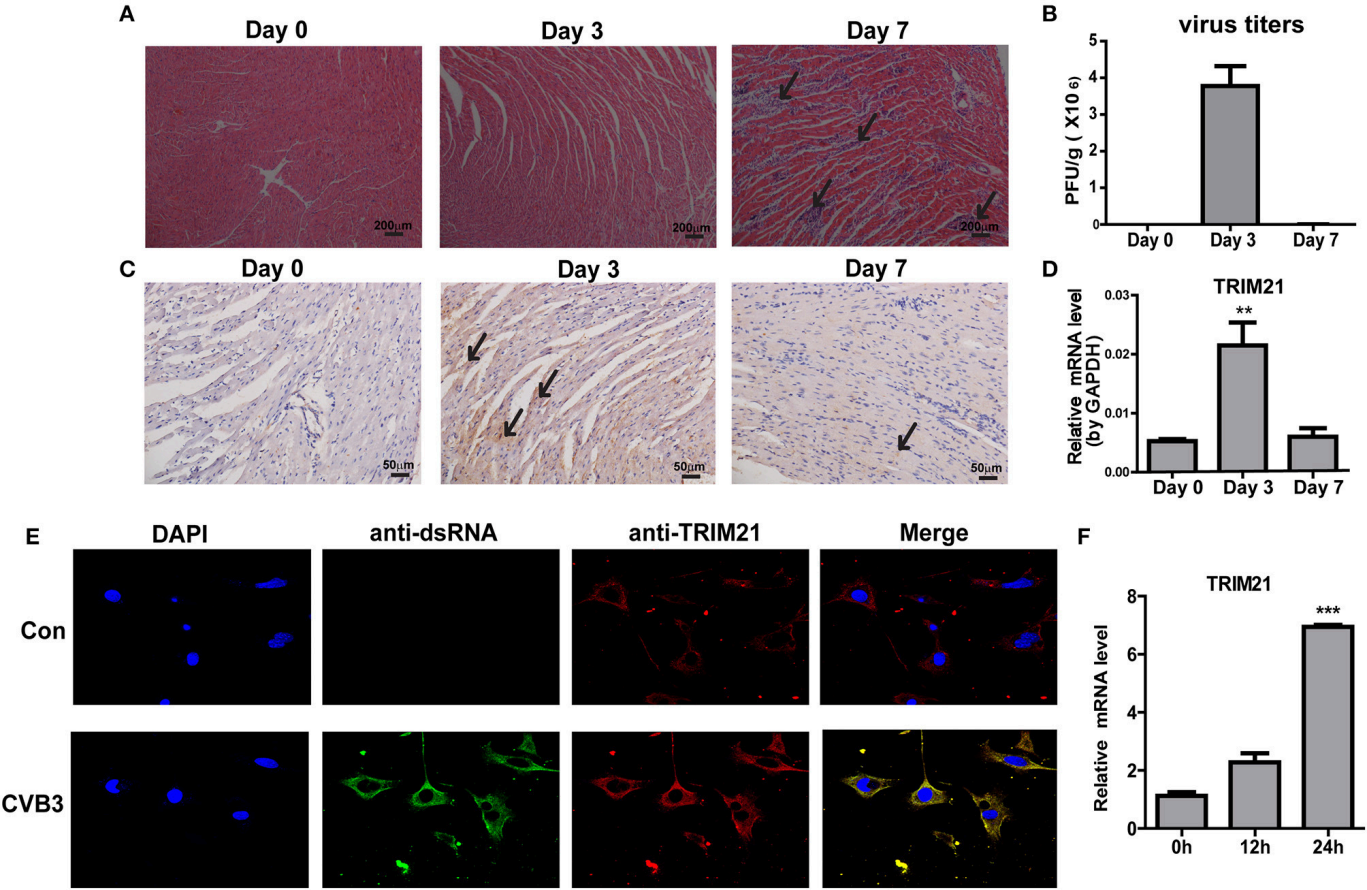
TRIM21 is up-regulated in heart tissues of CVB3-induced VMC mice. Male BALB/c mice were intraperitoneally injected with 1000 TCID_50_ dose of CVB3 and the tissues were collected at the indicated time. **(A)** Paraffin sections of heart tissues were prepared and subjected to H&E staining (200 × magnifications). **(B)** The viral titers were analyzed by TCID_50_ assay. Data were presented as mean ± SEM of three representative independent experiments. **(C)** TRIM21 protein level in heart tissues was evaluated by IHC assay. Five photomicrographs were captured at each time under high power fields (400× magnifications) randomly and one representative image was shown. **(D)** TRIM21 mRNA level was analyzed by Q-PCR. Data were presented as mean ± SEM of three representative independent experiments. The GAPDH expression levels of heart tissues was set 1.0. **(E,F)** Primary cardiomyocytes of mice were mock infected or infected by CVB3 (MOI = 5) for 24 h. CVB3 dsRNA, endogenous TRIM21 protein and nucleus were stained with anti-dsRNA antibody (green), anti-TRIM21Ab (red) and DAPI dye (blue) and observed under confocal microscope. Photomicrographs were captured under high power fields (100 × magnifications) **(E)**.TRIM21 mRNA level were analyzed by Q-PCR. Data were presented as mean ± SEM of three representative independent experiments. The GAPDH expression level of heart tissues was set as 1.0 **(F)**. **p < 0.01; ***p < 0.001.

To explore the role of TRIM21 in CVB3 infection, first we investigate whether TRIM21 is induced in heart tissues of mice by CVB3 infection. After 1000 TCID_50_ CVB3 i.p. infection, a massive inflammatory infiltration and cardiomyocye necrosis were observed in hearts at day 7 p.i. (Figure 1A). The viral load in myocardium increased and peaked at day 3 p.i., then declined at day 7 p.i. (Figure 1B). Then we detected the expression kinetics of TRIM21 by Q-PCR and immunohistochemistry. The protein and mRNA levels of TRIM21 in hearts of mice were significantly increased and peaked at day 3 p.i. (Figures 1C,D). To confirm the expression and localization of TRIM21 in cardiomyocytes, we cultured primary cardiomyocytes from newborn mice and infected cells with CVB3. The immunofluorescence assay showed that TRIM21 was localized in cytoplasm and CVB3 infection enhanced its expression at protein and RNA levels (Figures 1E,F).Therefore, our result demonstrate that cardiac TRIM21 expression is up-regulated by CVB3 infection, which may be involved in the regulation of CVB3 infection and the progression of viral myocarditis.

### TRIM21 suppresses CVB3 replication *in vitro*

**Figure 2:**
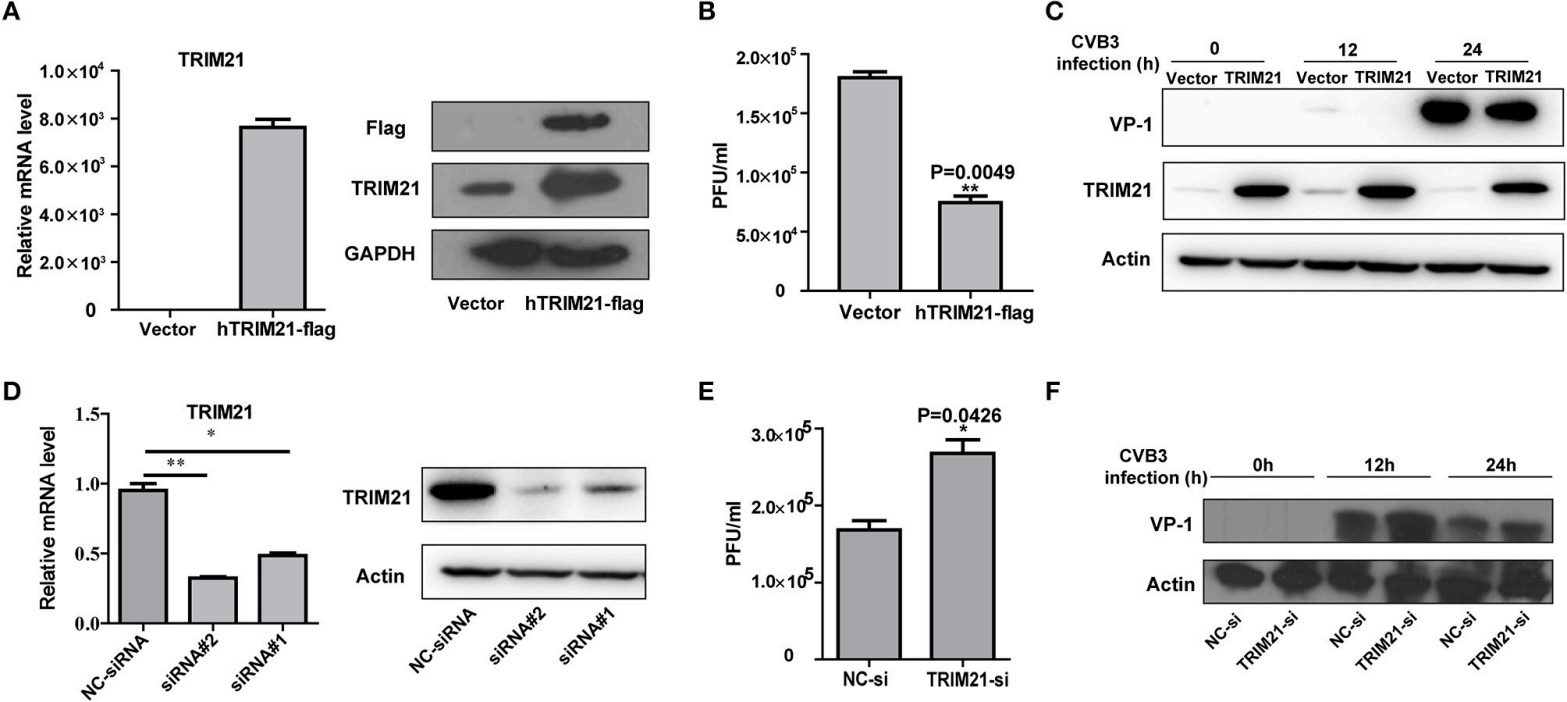
TRIM21 inhibits CVB3 infection *in vitro*. **(A)** HeLa cells were transiently transfected with human TRIM21-flag, or mock plasmids and 24 h later cells were subjected to Q-PCR and Western blot for detecting TRIM21 mRNA and protein levels. **(B–D)** HeLa cells were transiently transfected with human TRIM21-flag, or mock plasmids and 24 h later cells were infected with CVB3 at an MOI of 5 for the indicated time. Supernatants were collected and subjected to TCID_50_ **(B)**.Caspid protein of virus was analyzed by Western blot **(C)**. **(D)** HeLa cells were transfected with negative-control siRNA or TRIM21 siRNAs. 24 h later, cells were collected and subjected to Q-PCR and Western blot for analysis of TRIM21 expression.**(E,F)** HeLa cells were transfected with negative-control siRNA or TRIM21 siRNA and 24 h later, cells were infected with CVB3 (MOI = 5) for the indicated times. Supernatants were collected and subjected to TCID_50_ **(E)**. VP1caspid protein of virus were analyzed by Western blot **(F)**. *p < 0.05; **p < 0.01.

To investigate the role of TRIM21 on CVB3 replication, HeLa cells were transiently transfected with a plasmid expressing TRIM21 or vector alone and then infected with CVB3 at MOI of 5. The efficiency of overexpression of TRIM21 was confirmed by real-time PCR and Western blot (Figure 2A). The supernatant was subjected to TCID_50_ assay to determine the role of TRIM21 on viral progeny release. As shown in Figure 2B, TRIM21 overexpression significantly reduced the virus particle release. Furthermore, the protein level of CVB3 capsid VP1 was significantly inhibited by TRIM21 overexpression (Figure 2C).

To further verify the antiviral effect of TRIM21, we designed and screened two specific siRNA targeting the open reading frame of TRIM21, which led to a 75–80% reductions in the overall levels of the TRIM21 mRNA and protein (Figure 2D). Knockdown of TRIM21 increased CVB3 progeny production (Figure 2E) and viral capsid protein VP1 expression (Figure 2F) significantly, compared to the effect of NC siRNA. Collectively, these results confirm that TRIM21 significantly restricts CVB3 replication *in vitro*.

### TRIM21 increases IFN-α/β activation pathway

**Figure 3:**
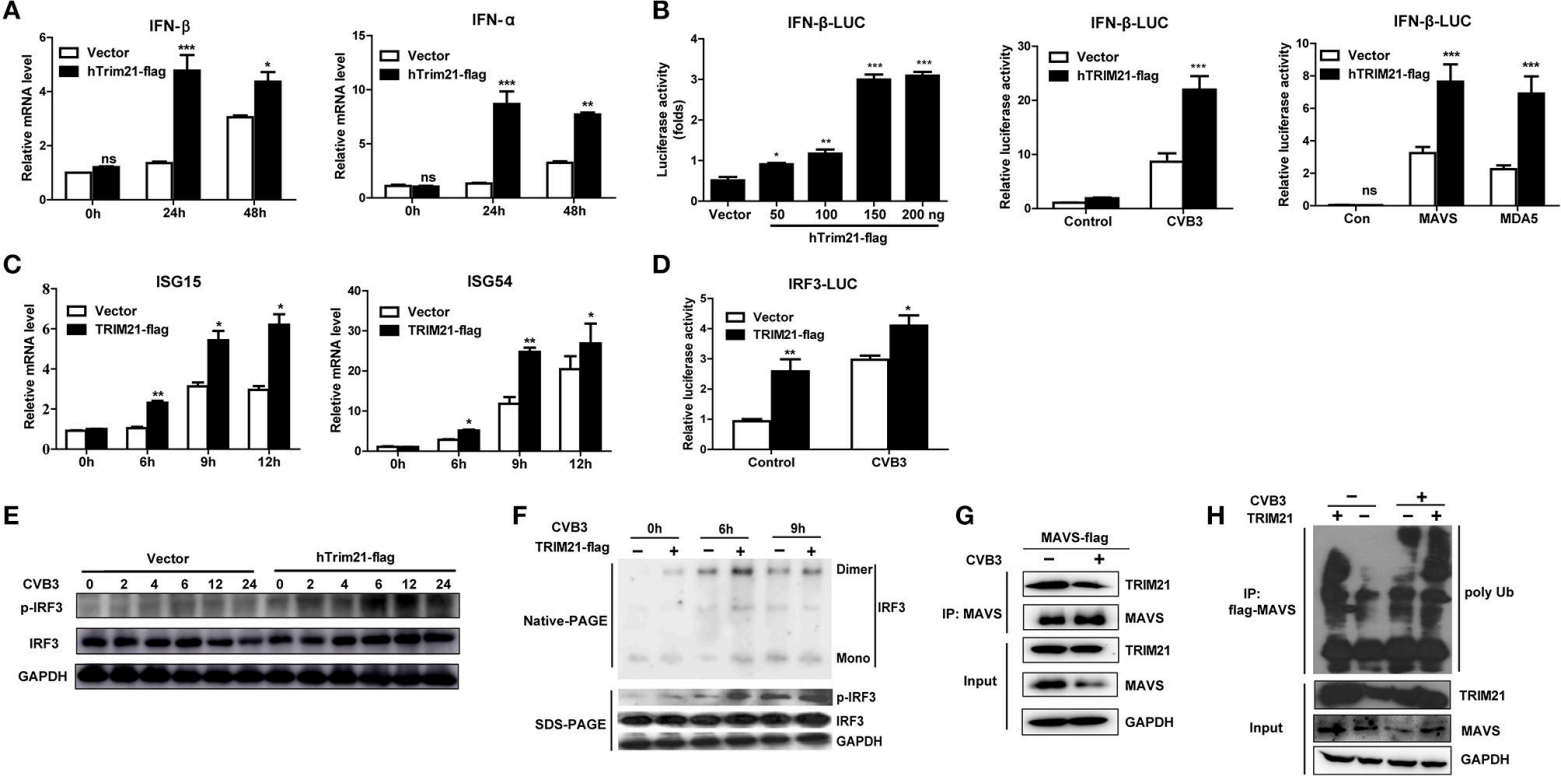
Overexpression of TRIM21 activates the virus-induced type I IFN signaling via catalyzing the K27-linked polyubiquitination of MAVS and increases IRF3 phosphorylation. **(A)** HeLa cells were transfected with TRIM21 expression or vector plasmids. 24 h after transfection, cells were infected with CVB3 (MOI = 5) for the indicated time. The mRNA levels of endogenous IFN-β and IFN-α were detected by Q-PCR assay. Data were presented as mean ± SEM of three representative independent experiments. **(B)** HEK293 cells were co-transfected with the IFN-β promoter reporter plasmids and the indicated amounts of TRIM21 expression plasmid or 100 ng vector plasmid. 24 h later, the luciferase activity was determined. HEK293 cells were co-transfected with the IFN-β promoter reporter plasmids and TRIM21 expression plasmid or mock vector. 24 h later, cells were infected with CVB3 (MOI = 5) for 12 h and the luciferase activity was determined. HEK293 cells were co-transfected with the IFN-β promoter reporter plasmids and TRIM21 expression plasmid or mock vector, together with MAVS or MDA5 expression plasmids. 24 h later, cells were infected with CVB3 (MOI = 5) for 12 h and the luciferase activity was determined. Data were presented as mean ± SEM of three representative independent experiments. **(C)** HeLa cells were transfected with TRIM21 expression or vector plasmids for 24 h before infection with CVB3 (MOI = 5). The mRNA levels of endogenous ISG15 and ISG54 were detected by Q-PCR assay. **(D)** HEK293 cells were co-transfected with the IRF3 promoter reporter plasmids and TRIM21 expression plasmid or mock vector. 24 h later, cells were infected with CVB3 (MOI = 5) for 12 h and the luciferase activity was determined and data were presented as mean ± SEM of three representative independent experiments. **(E,F)** HeLa cells were transfected with TRIM21 expression or vector plasmids for 24 h before infection with CVB3 (MOI = 5). Cell lysates were subjected to probe withanti-IRF3, anti-pIRF3 and anti-GAPDH antibodies by Western blotting and Native page for the detection of IRF3 dimerization. **(G)** HeLa cells were transfected with Flag-MAVS plasmids as indicated, 24 h later cells were infected with CVB3 (MOI = 5). Cellular lysates were immunoprecipitated with anti-Flag. Immunoprecipitates were analyzed by WB with anti-Flag and anti-TRIM21. **(H)** HeLa cells were co-transfected with TRIM21, Flag-MAVS, and HA-K27Ub for 24 h and treated with CVB3 for additional 12 h. Ubiquitination and immunoblotting assays were performed with indicated antibodies. *p < 0.05; **p < 0.01; ***p < 0.001.

Type I interferons (IFNs) play an important part in the resistance to viral infection. TRIM21 is reported to be involved in modulating host innate type I signaling against viral replication. Thus, we first examined the IFN-β mRNA production in HeLa cells overexpressing TRIM21 upon CVB3 infection by real-time PCR. Cells transfected with an empty vector were used as a control. In comparison to vector-transfect cells, a moderate promotion in IFN-β mRNA levels was observed in the TRIM21-overexpressed cells infected with CVB3. Additionally, IFN-α mRNA level was increased significantly by TRIM21 overexpression (Figure 3A). To confirm IFN-α/β promoting role of TRIM21, HeLa cells were co-transfected with TRIM21 vector and IFN-β promoter-luciferase plasmid. As demonstrated in Figure 3B, overexpression of TRIM21 enhanced the activity of IFN-β promoter in a dose-dependent manner after CVB3 infection. Furthermore, co-transfection of TRIM21 increased MAVS-activated and MAD5-activated IFN-β reporter gene expression. Next, we detected IFN-stimulated genes (ISGs) expression and found TRIM21 also up-regulated the expression of ISG15 and ISG54 upon CVB3 infection (Figure 3C). Thus, our data suggest that TRIM21 up-regulates the activation of IFN-β signaling pathway.

### TRIM21 positively regulates IRF3 activation via K27-linked polyubiquitination of MAVS upon CVB3 infection

Since TRIM21 promotes IFN-β activation after CVB3 infection, we suggest that TRIM21 might positively modulate the up-stream molecules of type I interferon signaling pathway. RIG-I and MDA-5 recognition of CVB3 RNA leads to the activation of IRF3 and transcription factors required for transcription activation of IFN-α/β. We next explored the effect of TRIM21 on IRF3 activation upon CVB3 infection. As shown in Figure 3D, overexpression of TRIM21 significantly enhanced the reporter activity of IRF3 at basal level and after CVB3 infection. Then, native page assay was performed and demonstrated that TRIM21 overexpression promoted the dimerization and phosphorylation of IRF3 (Figures 3E,F). Recently, Xue et al. reported that TRIM21 catalyzed the K27-linked polyubiquitination of MAVS to upregulate type-I interferons signaling upon RNA virus infection. So we examined the interaction between TRIM21 and MAVS. The CO-IP experiments revealed that TRIM21 interacted with MAVS while CVB3 infection reduced MAVS expression (Figure 3G). Degradation of MAVS by CVB3 pro2A (21) may counteract the effect of CVB3 on TRIM21 up-regulation and TRIM21-MAVS interaction *in vitro*. Furthermore, we observed that TRIM21 catalyzed the formation of K27-linked polyubiquitin chains on MAVS (Figure 3H, lane 1–2). Importantly, CVB3 infection enhanced the formation of the K27-linked polyubiquitinon MAVS by TRIM21 (Figure 3H, lane 3–4).Our data suggest that TRIM21 positively regulates type I IFN pathway during CVB3 infection via interacting with and promoting the ubiquitination of MAVS, thereby enhancing IRF3 activation.

### The ring and PRY-SPRY domains are required to facilitate the TRIM21-mediated anti-viral activity

**Figure 4:**
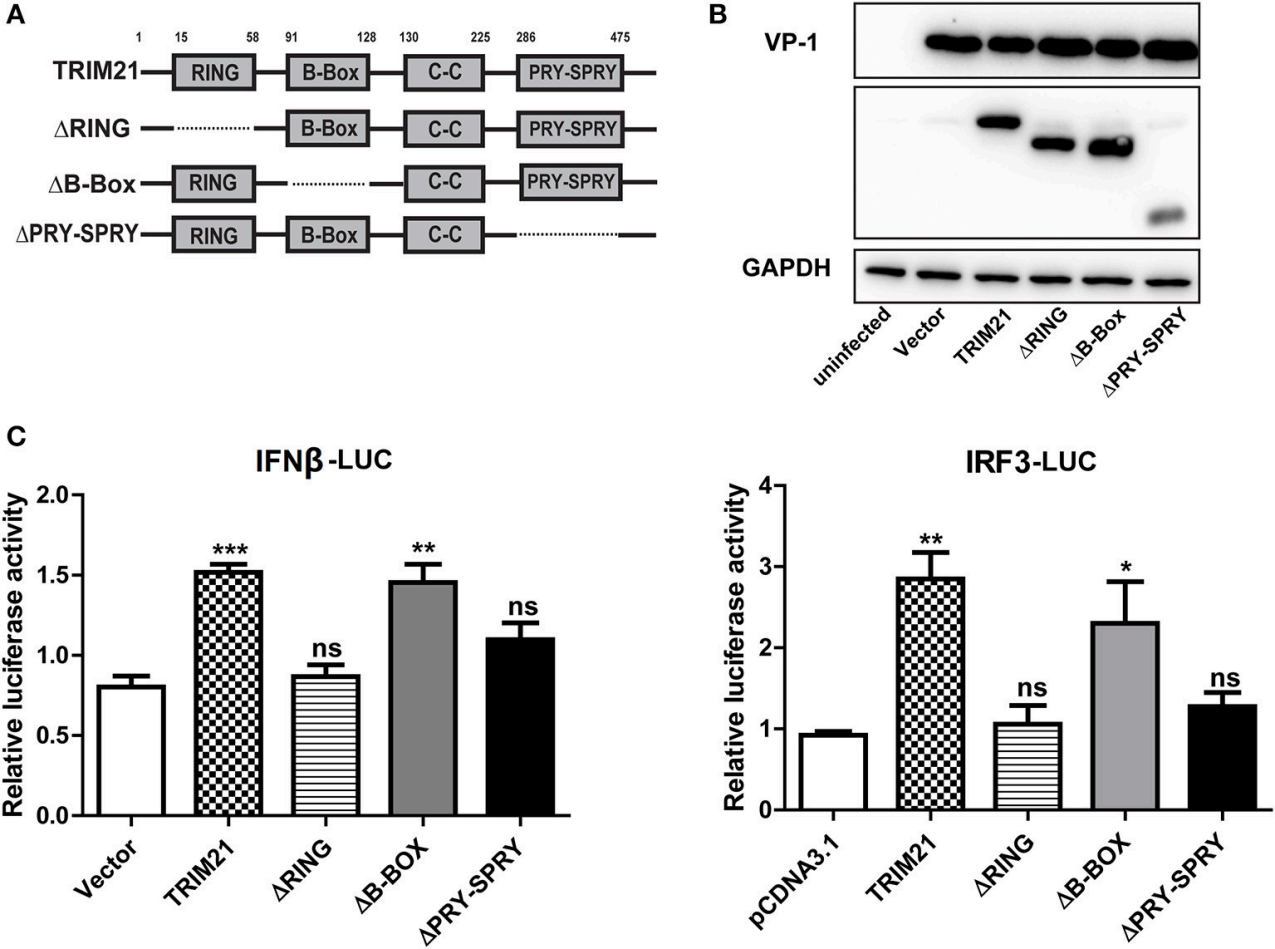
The RING domain and PRY-SPRY domain are essential for its antiviral effect against CVB3. **(A)** Schematic of domain organization and deletion mutants of TRIM21. Approximate amino acid positions of domains are shown at the top. Various domains are boxed and discontinuous lines represent deletion of those regions. **(B)** HeLa cells were transiently transfected with TRIM21 expression plasmid, or indicated domain deletion mutants and 24 h later cells were infected with CVB3 (MOI = 5) for 24 h. The expression efficiency of domain deletion mutants and VP-1 production were analyzed by Western blot. **(C)** HEK293 cells were transfected with the IFN-β or IRF3 promoter reporter plasmids, together with TRIM21 expression plasmid or the indicated domain deletion mutants. The luciferase activity was determined after 24 h and data were presented as mean ± SEM of three representative independent experiments. *p < 0.05; **p < 0.01; ***p < 0.001.

TRIM21 contains three classical motifs including a RING finger domain, a B-box domain and a B30.2 domain. We constructed various domain mutants of TRIM21 to define which part was involved in its antiviral role (Figure 4A). As compared with the full-length TRIM21, B-Box mutant showed similar anti-CVB3 effects, while RING and PRY-SPRY domain mutants abolished the antiviral effects as measured by western blot of viral VP-1 protein (Figure 4B). Furthermore, dysfunction of RING domain and PRY-SPRY domain obstructed the activating role of TRIM21 on the promoter of IFN-β and IRF3, while B-box mutant and B30.2 mutant had no effect (Figure 4C). Collectively, the RING domain with E3 ubiquitin ligase and the PRY-SPRY domain were required for the TRIM21-mediated type I IFN activation and anti-viral effect.

### *In vivo* overexpression of TRIM21 protects mice against CVB3-induced myocarditis

**Figure 5:**
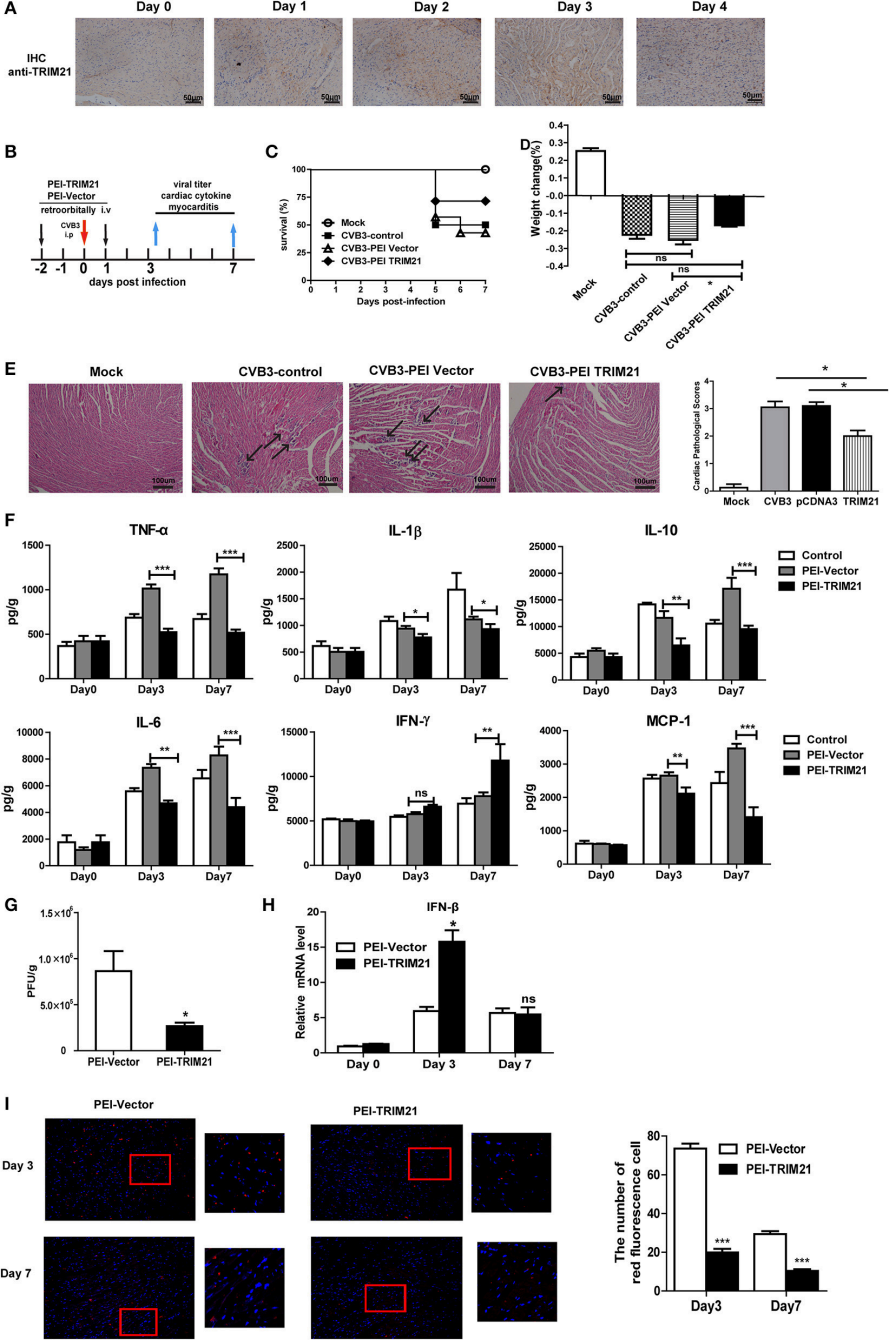
Overexpression of TRIM21 *in vivo* significantly reduces viral load and alleviates CVB3-induced viral myocarditis. **(A)** Male BALB/c mice were retroorbitally injected 50 μg TRIM21 plasmids using *in vivo*-jet PEI and were sacrificed daily till day 4. Protein level of cardiac TRIM21was measured by IHC assay. **(B)** Mice were injected retroordibitally with 2 doses of 50 μg PEI-packaged mock or TRIM21 plasmids on day −2 and 1 and subjected to 1000TCID_50_ CVB3 on day 0(*n* = 6). The survival rate **(C)** and body weight change **(D)** were monitored daily until day 7 p.i. **(E)** Representative image of HE-staining hearts of CVB3-infected mice (day 7 p.i.) treated with PEI-TRIM21 or PEI-vector, showing intra-cardiac immune infiltrates (marked with arrows). Scale bar: 100 μm. Pathological scores of the heart of mice are shown. Results are presented as mean ± SEM; ^*^p < 0.05. **(F)** Protein levels of inflammatory cytokines in the homogenates of heart were measured by ELISA. Data were presented as mean ± SEM of three representative independent. **(G)** The cardiac CVB3 titer at day 3 p.i. were determined by TCID_50_ assay. Data represent mean values of CVB3 PFU per gram of the heart tissues. Results are presented as mean ± SEM; Data pooled from 3 independent experiments. ^*^p < 0.05; ^**^p < 0.01. **(H)** Relative mRNA level of IFN-β (day 3 and 7p.i.) was detected by Q-PCR. Data were normalized to GAPDH expression and presented as mean ± SEM of three representative independent. **(I)** Hearts of mice at day 3 and 7 p.i. were OCT-embedded and cyrosections (5 μM) were subjected to fluorescent staining. Composite confocal represented images show dsRNA (red, anti-dsRNA Ab) and nuclear (blue, DAPI).Low magnification (magnification, × 100) and higher magnification of the boxed areas (magnification, × 200) are shown. The number of red-stained viral-infected cells in the heart sections of mice were numerated. Data are expressed as mean ± SEM from three repeated experiments (*n* = 3). ***p < 0.001.

We next evaluate the antiviral effect of TRIM21 *in vivo* according to an *in vivo*-JetPEI^TM^ strategy (22) and one retroorbital injection of 50 μg TRIM21- plasmids led to an enhanced protein expression of cardiac TRIM21 which sustained for 2–3 days confirmed by IHC analysis (Figure 5A). Therefore, groups of mice were retro-orbitally injected with 50 μg TRIM21-plasmids or vector-plasmids using *in vivo*-Jet PEI reagent 2 days before and 1 day after CVB3 infection (Figure 5B), and susceptibility to CVB3 myocarditis as well as viral replication were evaluated in a course of 7 days infection. The survival rate and bodyweight loss of mice were monitored by day 7 p.i. and a significantly improved disease condition and reduced mortality were observed in mice with *in vivo* TRIM21 overexpression. More than 60% mice injected with mock plasmids died by day 7 and lost their 28% bodyweight; while TRIM21-overexpressed mice underwent a gentle decline loss of bodyweight (~17%) and ~70% mice survived by day 7 p.i. (Figures 5C,D). Consistently, histological analysis revealed that mice with mock plasmids developed severe myocarditis with diffuse inflammation and necrotic lesions, whereas TRIM21 treatment attenuated myocarditis with restricted inflammation and necrosis (Figure 5E). By analyzing the levels of cardiac inflammatory cytokines we found that inflammatory cytokines, such as IL-1β, TNF-α, IL-6, IL-10, and MCP-1, were significantly reduced by TRIM21 overexpression while IFN-γ level was not affected (Figure 5F).

To test whether differences in CVB3 disease susceptibility were due to differences in viral titers, CVB3 burden in the hearts of mice were measured. As shown in Figure 5G, CVB3 titer was significantly reduced in hearts of mice with TRIM21 over-expression at day 3 p.i. Immunofluorescent staining of the heart sections also confirmed dramatically reduced viral RNA level in hearts (Figure 5I). In accordance with that, a significantly up-regulated mRNA expression of IFN-β in heart was confirmed in TRIM21 overexpressed mice at day 3 p.i.(Figure 5H).

### TRIM21 deficient mice exhibits increased cardiac and pancreatic viral burden and aggravated myocarditis as well as pancreatic necrosis

**Figure 6:**
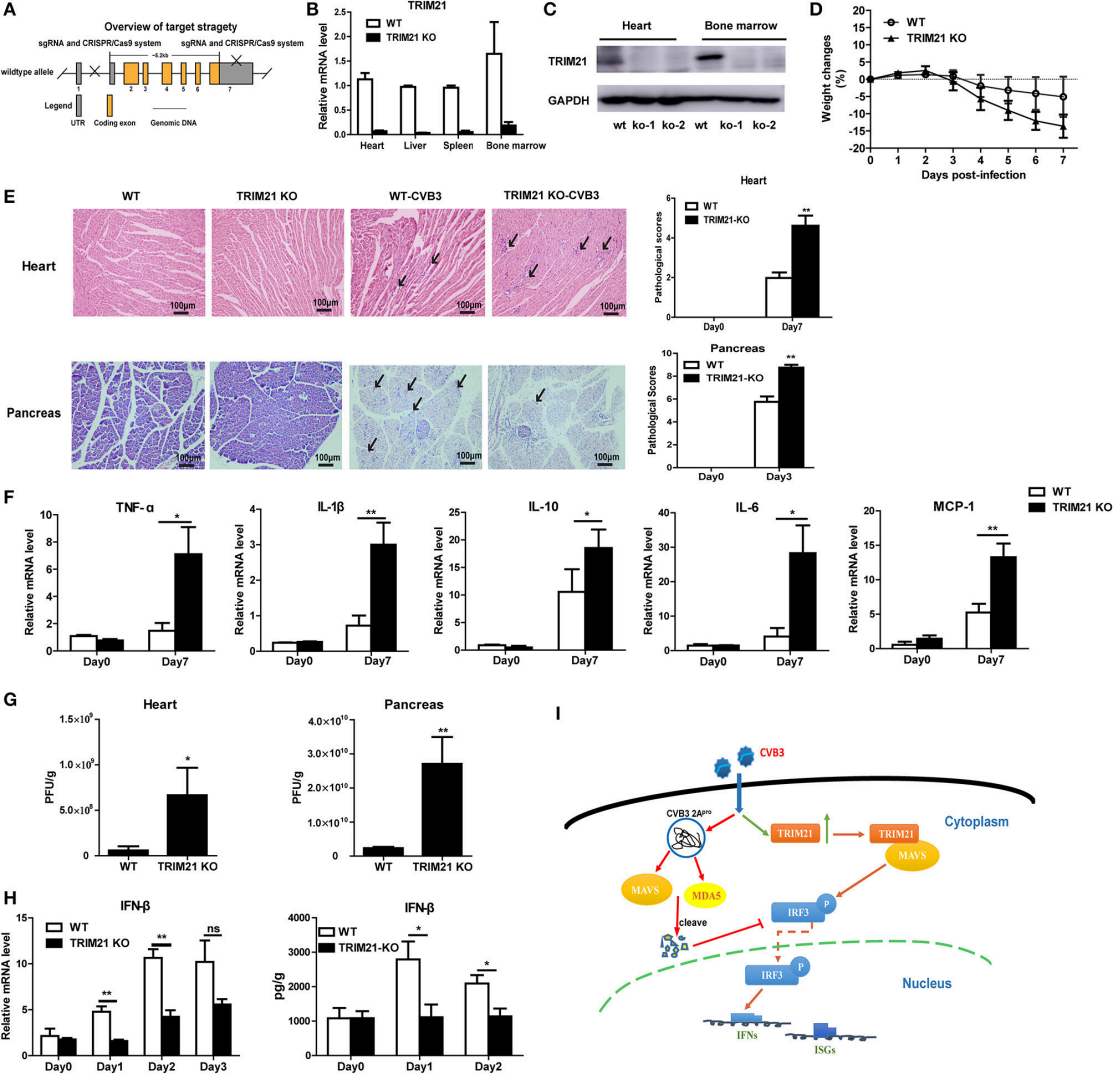
TRIM21 deficient mice increases CVB3 replication in organs and aggravates pancreatic acinar cell necrosis as well as myocarditis. **(A)** Schematic diagram of deficient mice construction by CRISPR-CAS9 strategy. **(B,C)** Q-PCR and Western blot analysis of TRIM21 expression from tissues and BM cells of wild-type (WT) and TRIM21-/- mice, GAPDH was used as a loading control. **(D-H)** WT and TRIM21-deficient mice were infected i.p. with CVB3 (*n* = 6).The body weight change were monitored daily until day 7 p.i. **(D)**. Representative image of HE-staining hearts and pancreas of CVB3-infected WT or TRIM21-/-mice (day 7 p.i.), showing intra-cardiac immune infiltrates or intactpancreatic acini (marked with arrows). Scale bar: 100 μm. Pathological scores of the heart and pancreas of mice are shown. Results are presented as mean ± SEM; Data pooled from 3 independent experiments **(E)**.The mRNA levels of inflammatory cytokines in the homogenates of heart (day 7 p.i.) were measured by Q-PCR. Data were presented as mean ± SEM of three representative independent **(F)**.Viral loadin pancreas and hearts of mice(day 3p.i.) was assessed by TCID_50_ assay. Results are presented as mean ± SEM;Data pooled from 3 independent experiments. *p < 0.05; **p < 0.01 **(G)**.The mRNA and protein level of IFN-β (day 1–3 p.i.) in hearts of mice was detected by Q-PCR and ELISA. Data as mean ± SEM of three representative independent. *p < 0.05; **p < 0.01 **(H)**. **(I)** Proposed model depicting the role of TRIM21 in positive regulation of IFN-I production during CVB3 infection. TRIM21 targets and promotes the activity of MAVS, leading to the increased phosphorylation and translocation of p-IRF3 into the nucleus, leading to enhanced transcription and production of IFNs and IFN-stimulated genes (ISGs) that limits CVB3 infection.

To further confirm the antiviral effect of TRIM21 *in vivo*, we constructed deficient mice by CRISPR-CAS9 strategy (Figure 6A) and confirmed the deletion of mRNA and protein level of TRIM21 in tissues of mice (Figures 6B,C). Next, WT and TRIM21-deficient mice were infected i.p. with CVB3. Throughout the 7 days infection, TRIM21-deficient mice exhibited greater signs of sickness at day3 p.i. and lost weight more promptly by day 7 p.i. (14.8 vs. 5.1%, TRIM21-deficient vs. WT, *p* < 0.05, Figure 6D). Histopathology analysis revealed that TRIM21-deficient mice exhibited a significantly aggravated coagulative necrosis and acinar cell necrosis in the pancreas at day3 p.i., and an increased cardiac immune infiltration at day 7 p.i. (Figure 6E) compared to WT mice. Consistent with enhanced tissue pathology, the levels of cardiac inflammatory cytokines were significantly increased in TRIM21 KO mice than in WT mice (Figure 6F).To test whether differences in CVB3 disease susceptibility were due to differences in viral replication, CVB3 burden in the hearts and pancreas of mice were measured. At 3 dpi, the peak of viral replication, TRIM21 deficient mice exhibited significantly increased viral titers in hearts and pancreas compared to WT mice (Figure 6G). To confirm the anti-viral effect of TRIM21 *in vivo*, the mRNA and protein level of IFN-β in hearts were measured and were found significantly decreased in TRIM21 deficiency mice at early infection stage compared to those in WT mice (Figure 6H). These data confirm that TRIM21 effectively suppresses CVB3 replication *in vivo*. We thus propose a model depicting the role of TRIM21 in CVB3 infection: CVB3 infection up-regulates the expression of TRIM21 in cardiomyocytes, which interacts with MAVS and promotes IRF3-mediated IFN-I signaling to suppress viral replication *in vivo*, thereby decreasing virus-induced inflammatory injury in both hearts and pancreas of mice (Figure 6I).

## Discussion

In this study, we try to explore the role of TRIM21 in the susceptibility of mice to CVB3 induced myocarditis. TRIM21 expression is significantly up-regulated in hearts of mice on day 3 post infection, and systemic TRIM21 effectively inhibits CVB3 replication *in vivo*. TRIM21 restricts CVB3 replication by positively regulating IRF3 activation and IFN-β production after CVB3 infection via interacting with and promoting K27-linked polyubiquitination of MAVS. Silencing of TRIM21 significantly enhances CVB3 replication in tissues and alleviates virus-induced cardiac and pancreatic injury. Treatment with CVB3-infected mice with TRIM21 significantly reduces CVB3 replication in hearts and the severity of viral myocarditis.

TRIM21, initially known as an autoantigen Ro52/SS-A, is an ubiquitously expressed cytosolic E3 ubiquitin ligase and plays important roles in immune regulation and microbial restriction (23, 24). It has been reported that TRIM21 is constitutively and broadly expressed in various organs and cell types, but with highly divergent levels of expression. Highest expression is seen in cells of the immune system with particularly high levels in T cells, macrophages and DCs, where the expression is further augmented by stimulation with IFNs and TLR ligation (25). Previous study finds that TRIM21 expression is substantially increased in human primary lymphocytes and monocyte-derived macrophages in response to interferons (IFNs, type I and II), suggesting TRIM21 as an interferon-induced gene (26). It has been reported that SeV, NDV or HCV infection could significantly induce the expression of TRIM21 through JAK/STAT signaling pathway (27). Thus, although CVB3 infection does not induce robust production of IFN-β, a significant induction of TRIM21 protein is observed in heart tissues upon CVB3 infection. More convincingly, TRIM21 expression is significantly enhanced in primary cardiomyocytes upon CVB3 infection (Figures 1E,F) which is localized in cytoplasm of cells.

The host cells activate a series of signaling events that lead to induction of type I interferons (IFNs), including IFN-β and IFN-α. Type I IFNs further induce the expression of downstream proteins, which mediate innate immune responses, such as suppression of viral replication, clearance of virus-infected cells (28, 29). The role of TRIM21 in regulating the type I interferon signaling has been controversial. Sunit K Singh demonstrates TRIM21 as a negative regulator of IFN-β production mediated by IRF-3 during JEV infection in human microglial cells (19). In 2015, another study finds that TRIM21 facilitates Nmi-mediated negative regulation of the innate antiviral response (32). And Liu Y's group reports that TRIM21 as an E3 ligase which induces the Lys48 (K48)-linked ubiquitination and degradation of DDX41 and negatively regulates the innate immune response to intracellular dsDNA in myeloid dendritic cells (30). All the above data indicate that TRIM21 is a negative regulator of IRF3 (or DD41) activation and IFN-β production. However, Chen Wang reports that TRIM21 is induced and interacts with IRF3, preventing IRF3 ubiquitination and degradation thus playing an anti-viral effect during SeV infection (17). Recently, Xue et.al have reported (20) that TRIM21 is upregulated upon RNA virus (SeV, VSV) infection, interacts with MAVS and catalyzes the K27-linked polyubiquitination of MAVS, thereby promoting the activation of IRF3 and inhibiting viral infection. In our study, we confirm the co-immunoprecipitation and polyubiquitination of TRIM21 with MAVS (Figures 3G,H), which is in consistency with Xue's report that TRIM21 interacts with MAVS and promotes K27-linked polyubiquitination of MAVS. It also supports our conclusion that the anti-viral effect of TRIM21 is RING domain dependent (Figure 4C). Finally we propose a model depicting the role of TRIM21 in CVB3 infection: CVB3 infection up-regulates the expression of TRIM21 in mice, TRIM21 interacts with MAVS and promotes the activation of IRF3 resulting in an up-regulation of type I innate signaling during CVB3 infection (Figure 6I).Although MDA5, MAVS and RIG-I are cleaved by CVB3 2Apro and 3Cpro (21) indicating TRIM21, one of ISGs, might be hardly up-regulated during CVB3 infection and TRIM21-mediated IFN-I response enhancing effect might be counteracted, there is article suggesting that enhancing IFNs production might be an alternative prescription in CVB3-related syndromes (33). And our *in vivo* over-expression and deficiency experiment in mice confirm TRIM21 supplementation as a promising strategy to limit CVB3 infection and related cardiac and pancreatic pathology.

CVB3 has evolved many strategies to suppress host innate immunity therefore does not cause robust interferon release and ISG expression. As shown in Figure 3A, the mRNA expressions of IFN-α and IFN-β in cells at 24 h after CVB3 infection were quite low (as similar as the level seen before infection). 48 h after infection, CVB3 did not induce IFN-α expression while induced a very modest up-regulation of IFN-β. Only upon transfection with TRIM21 plasmid, the mRNA expressions of IFN-α/β were significantly up-regulated at both 24 and 48 h post infection. The induced up-regulation of ISG15 by CVB3 was also very moderate, only TRIM21 overexpression significantly promoted ISG15 expression. It seems that upregulation of IFN-α/β may be a TRIM21-mediated effect. However, our data demonstrate that CVB3 infection significantly increases expression of TRIM21 in hearts of mice (Figures 1 A–D) and in primary cardiomyocytes (Figures 1E,F). Therefore, the upregulation of IFN-α/β signaling by TRIM21 is at least partially dependent on CVB3 infection. And the interaction of TRIM21 with MAVS (Figures 3G,H) further supports our data that CVB3-induced TRIM21 could enhance the phosphorylation of IRF3 upon CVB3 infection leading to elevated IFN-β production.

As a member of tripartite motif (TRIM) family protein, TRIM21 contains a RNIG motif in the N-terminal domain, a B-box motif, a coiled-coil domain. And TRIM21 protein also contains a carboxy-terminal B30.2 (SPRY) domain (31). Previous study demonstrates that TRIM21 interacts with IRF3 directly via its C-terminal SPRY domain, resulting in the polyubiquitination and proteasomal degradation of IRF3 and reduced IFN-β promoter activity (18). Liu Y's group report that TRIM21 cannot interact with IRF3 in mDC but interact with DD41 for promoting the ubiquitination and degradation of DDX41 therefore negatively regulates IFN-I response to DNA virus (HSV) (30). In 2015, another study finds that during SeV and VSV infection, up-regulated TRIM21 interacts with both Nmi and IFI35 and activates K63-linked ubiquitination on K22 residue of Nmi (SPRY domain dependent) which facilitates the negative regulatory function of the Nmi-IFI35 complex on innate antiviral signaling (32). Yang et al. report in their study that upon RNA virus infection TRIM21 interacts with IRF3, interferes with the interaction between Pin1 and IRF3, thus preventing IRF3 ubiquitination and degradation via its B30.2 domain (17). Xue et al. reports that TRIM21 interacts with MAVS and catalyzes the K27-linked polyubiquitination of MAVS through its RING domain (20). In our study, we confirmed (Figure 3G) that TRIM21 interacts with and promotes the K27-ubiquitination of MAVS, and the anti-viral effect of TRIM21 is RING and PRY-SPRY domain dependent (Figure 4), which is in consistency with the recent report (20).

Currently, there is only limited report of antiviral effect of TRIM21 in mice model. Our study demonstrate the *in vivo* effect of TRIM21 on CVB3 replication and tissue pathology. By using *in vivo*-Jet PEI-transfection of TRIM21-plasmids and TRIM21 deficient mice, we demonstrated that the viral replication and CVB3-induced cardiac immune infiltration, cardiac proinflammatory cytokines production and injuries were significantly decreased upon *in vivo* over-expression of TRIM21 (Figure 5). In accordance with that, cardiac and pancreatic CVB3 replication as well as virus-induced pancreatic acinar cell necrosis and myocarditis were significantly aggravated in TRIM21 deficient mice (Figure 6). Our data identify TRIM21 as a potent viral inhibitory factor during CVB3 infection. Recently, TRIM21 is also identified as an intracellular Fc receptor linking cytosolic antibody recognition to the ubiquitin proteasome system (34–36).So we cannot rule out the antiviral effect of TRIM21 is partially dependent on antibody-dependent intracellular neutralization (ADIN) effect of TRIM21 *in vivo*. And our preliminary data show that TRIM21 has IgA-mediated ADIN effect on CVB3 replication *in vitro* (data not shown). So in further study we will focus on clarifying whether TRIM21 exerts IgA-mediated ADIN function on intestinal CVB3 replication considering CVB3 as an oral-fecal disseminating virus.

Overall, our study identifies cytosolic TRIM21 as a positive regulator of CVB3-triggered MAVS-mediated type I Interferon signaling pathway that restricts viral infection. TRIM21 expression is up-regulated by CVB3 infection at early phase of viral infection. TRIM21 inhibits CVB3 replication *in vivo* and *in vitro* through interacting with MAVS thereby promoting the activation of IRF3 and Type I Interferon production. The anti-viral effect of TRIM21 is dependent on RING and PRY-SPRY domain. We also demonstrate the antiviral effect of systemic TRIM21 *in vivo* which leads to the increased resistance to CVB3-induced myocarditis and pancreatic injury. Our data help to clarify the biological role of TRIM21 in severe tissue pathology caused by viral infection and indicating a therapeutic target potential for TRIM21.

## Ethics statement

All animal experiments were performed in accordance with Soochow University institutional guidelines, and the study was approved by the Ethics Committee of Soochow University in written form (SYXK2015-0036).

## Author contributions

WX conceived and supervised the project. HL and YS performed the experiments. HL and ML interpreted data and wrote the manuscript. All authors approved the final version of the paper.

### Conflict of interest statement

The authors declare that the research was conducted in the absence of any commercial or financial relationships that could be construed as a potential conflict of interest.
